# Genomic sequencing of Troides aeacus nucleopolyhedrovirus (TraeNPV) from golden birdwing larvae (*Troides aeacus formosanus*) to reveal defective *Autographa californica* NPV genomic features

**DOI:** 10.1186/s12864-019-5713-2

**Published:** 2019-05-27

**Authors:** Yu-Feng Huang, Tzu-Han Chen, Zih-Ting Chang, Tai-Chuan Wang, Se Jin Lee, Jong Cheol Kim, Jae Su Kim, Kuo-Ping Chiu, Yu-Shin Nai

**Affiliations:** 1 Genomics Research Center, Academia Sinica, Taipei, Taiwan; 2 Department of Biotechnology and Animal Science, National Ilan University, Yilan, Taiwan; 3 Department of Entomology, National Taiwan University, Taipei, Taiwan; 4 Department of Agricultural Biology, College of Agriculture & Life Sciences, Chonbuk National University, Jeonju, South Korea; 5 Department of Entomology, National Chung Hsing University, Taichung, Taiwan

**Keywords:** *Troides aeacus*, *Troides aeacus* nucleopolyhedrovirus, TraeNPV

## Abstract

**Background:**

The golden birdwing butterfly (*Troides aeacus formosanus*) is a rarely observed species in Taiwan. Recently, a typical symptom of nuclear polyhedrosis was found in reared *T. aeacus* larvae. From the previous Kimura-2 parameter (K-2-P) analysis based on the nucleotide sequence of three genes in this isolate, *polh*, *lef*-*8* and *lef*-*9*, the underlying virus did not belong to any known nucleopolyhedrovirus (NPV) species. Therefore, this NPV was provisionally named “TraeNPV”. To understand this NPV, the nucleotide sequence of the whole TraeNPV genome was determined using next-generation sequencing (NGS) technology.

**Results:**

The genome of TraeNPV is 125,477 bp in length with 144 putative open reading frames (ORFs) and its GC content is 40.45%. A phylogenetic analysis based on the 37 baculoviral core genes suggested that TraeNPV is a Group I NPV that is closely related to *Autographa californica* nucleopolyhedrovirus (AcMNPV). A genome-wide analysis showed that TraeNPV has some different features in its genome compared with other NPVs. Two novel ORFs (*Ta75* and *Ta139*), three truncated ORFs (*pcna*, *he65* and *bro*) and one duplicated ORF (*38.7 K*) were found in the TraeNPV genome; moreover, there are fewer homologous regions (hrs) than there are in AcMNPV, which shares eight hrs within the TraeNPV genome. TraeNPV shares similar genomic features with AcMNPV, including the gene content, gene arrangement and gene/genome identity, but TraeNPV lacks 15 homologous ORFs from AcMNPV in its genome, such as *ctx*, *host cell-specific factor 1* (*hcf-1*), *PNK/PNL*, *vp15,* and *apsup*, which are involved in the auxiliary functions of alphabaculoviruses.

**Conclusions:**

Based on these data, TraeNPV would be clarified as a new NPV species with defective AcMNPV genomic features. The precise relationship between TraeNPV and other closely related NPV species were further investigated. This report could provide comprehensive information on TraeNPV for evolutionary insights into butterfly-infected NPV.

**Electronic supplementary material:**

The online version of this article (10.1186/s12864-019-5713-2) contains supplementary material, which is available to authorized users.

## Background

The golden birdwing butterfly, *Troides aeacus formosanus* (Rothschild) (Lepidoptera: Papilionidae), is one subspecies of five known *T. aeacus*; it is distributed throughout tropical areas and is also endemic to Taiwan [1]. Golden birdwing butterflies have a large body size and a wingspan that exceeds 15 cm [2]. The population of the golden birdwing butterfly has been declining due to commercial activity and a loss of habitat fitness, i.e., a loss of host plants [1, 3]. Therefore, this butterfly species is protected by the Convention on International Trade in Endangered Species of Wild Fauna and Flora (CITES), and the public will have to make more effort in the conservation management of the *T. aeacus formosanus* population [1]. From our previous investigation, a liquefaction symptom was found in the population of rearing golden birdwing butterfly larvae, and this symptom was similar to that of nuclear polyhedrosis. Polyhedral inclusion bodies (PIBs) were observed, and they filled in the body fluid of moribund larvae. A positive signal indicating a polyhedrin gene fragment was detected by PCR. Apparently, the polyhedrosis of the golden birdwing butterfly larvae is caused by nucleopolyhedrovirus (NPV) infection [4].

There are four genera in the *Baculoviridae*, including *Alphabaculovirus* (lepidopteran-specific nucleopolyhedrovirus, NPV), *Betabaculovirus* (lepidopteran-specific granulovirus), *Gammabaculovirus* (hymenopteran-specific NPV) and *Deltabaculovirus* (dipteran-specific NPV) [5]. The phylogenetic analysis based on the polyhedrin (*polh*) genes could further divide the lepidopteran-specific NPVs into group, I and II [6]. To date, more than 78 complete NPV genomes have been deposited in the NCBI GenBank, and most of them are lepidopteran-specific NPVs. However, the occurrence of NPV epizootics in butterfly species is uncommon. Among these sequenced NPV genomes, only *Catopsilia pomona* NPV (CapoNPV) was reported as a butterfly-infecting NPV, and it was clarified as a distinct species in Group I *Alphabaculovirus* [7].

To understand the NPV from the golden birdwing butterfly larvae, the Kimura 2-parameter (K-2-P) distances between the alignment of the *polh*, *lef-8* and *lef-9* nucleotide sequences were performed as described by Jehle et al. for baculovirus identification and species classification [8]. According to the analysis of K-2-P distances from these three genes, this NPV belongs to the group I baculoviruses and is highly closely related to the *Autographa californica* nucleopolyhedrovirus (AcMNPV) group [4]. However, most of the distances between this NPV and other closely related NPVs were higher than 0.015. The K-2-P results also showed an ambiguous taxonomic position for this virus; therefore, the taxonomic status of this virus still requires further clarification. Thus far, we could conclude that this NPV belongs to neither the BmNPV group nor the AcMNPV group*.* Therefore, this NPV was provisionally named “TraeNPV” [4].

As aforementioned, we attempted to sequence the whole genome of TraeNPV. Furthermore, a phylogenetic analysis based on 37 baculovirus core genes of 77 sequenced baculoviruses will be analysed to clarify the TraeNPV taxonomic issue. The genomic features of the whole genome, including the gene structure, orientations and genome density will be described in this report. Comparative genomic analyses were also performed, and the genome sequences were further compared in detail with the previously published group I NPV type species including AcMNPV [9], *Bombyx mori* NPV (BmNPV) [10], *Maruca vitrata* MNPV (MaviMNPV) [11], group II NPV type species LdMNPV [12] and one Betabaculovirus, the *Cydia pomonella* granulosis virus (CpGV) [13]. This report provides new insight into evolutionary aspects of butterfly-infecting NPVs. Therefore, the precise relationship between TraeNPV and other closely related NPV species could be further investigated.

## Results and discussion

### General characteristics of the TraeNPV genome

**Fig. 1:**
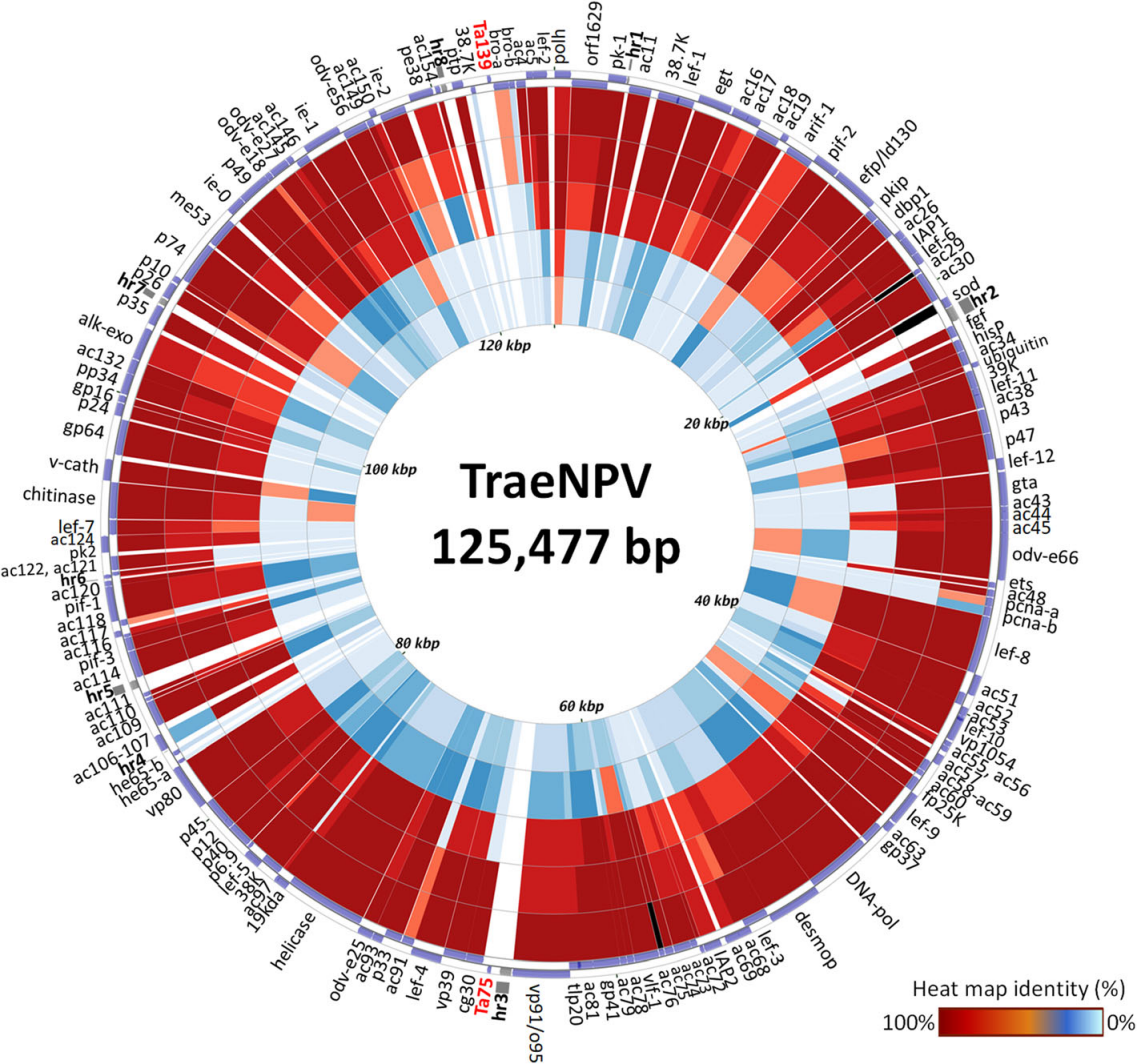
Genomic circular map and heat map identity of the TraeNPV. The heat map identity of the species AcMNPV, BmNPV, MaviMNPV, LdMNPV and CpGV compared to the orthologous ORFs of TraeNPV are shown on the inner rings in sequence. The darker the red is, the higher the correlated ORF identity. The positions for these 144 ORFs, which are listed in Additional file 1: Table S2, are presented as arrowheads with the direction of the arrowhead indicating the orientation of each ORF. The locations for the eight homologous repeat regions (hrs) are indicated

The TraeNPV genome is 125,477 bp in length and has a G + C content of 40.35% (see Additional file 1: Table S1). The complete genomic sequence with gene annotation information was submitted to GenBank (accession number: MH077961). The open reading frames (ORFs) were predicted according to the initial criteria for further study. A total of 144 ORFs were identified for further analysis (Fig. 1; Additional file 1: Table S2), and the nucleotides in the TraeNPV genome were numbered sequentially, beginning with the A (designated position 1) of the *polyhedrin* start codon (ATG). The arrows indicate the directions of the transcripts. The ratio of the ORF orientations was approximately 1:1.06 [clockwise (70/144): anticlockwise (74/144)] for those oriented clockwise with respect to the orientation of the *polh* gene (ORF1) [14]. The TraeNPV genome had a high number of ORFs, which ranked 18.99% (15/79) compared to the other 78 sequenced baculovirus genomes (Additional file 2: Figure S1). Among these putative ORFs, 40.97% (59 ORFs) showed overlap in the genome, and the length of the overlap ranged from 1 bp to 158 bp. Four pairs of ORFs that had a larger overlap than that found in TraeNPV were identified, namely, *Ta59* (*lef-3*)/*Ta60* (*ac68*), *Ta72* (*ac81*)/*Ta73* (*tlp20*), *Ta106* (*ac121*)/*Ta107* (*ac122*) and *Ta5* (*38.7 K*)/*Ta6* (*lef-1*). *Ta59* overlaps with *Ta60* by 52 aa in the opposite ORF direction. *Ta72* overlaps with *Ta73* by ca. 50 aa. There were ca. 36 aa of overlap between the ORFs *Ta106*/*Ta107* and *Ta5*/*Ta6*. There were 37 conserved genes in all the baculovirus genomes, including the dipteran and hymenopteran baculoviruses [15–18], and all of these genes were found in the TraeNPV genome. Except in the TraeNPV genome, the *Ac108* was found in all the alpha- and betabaculovirus genomes [19]. Moreover, two baculovirus-repeated ORFs (the *bro* genes *bro-a* and *bro-a*) were also identified in this genomic sequence. Most of the 144 TraeNPV ORFs had related homologues in other baculoviruses except for two unique ORFs (*Ta75* and *Ta139*), which were identified in the TraeNPV genome (Fig. 1; Additional file 1: Table S2).

**Fig. 2:**
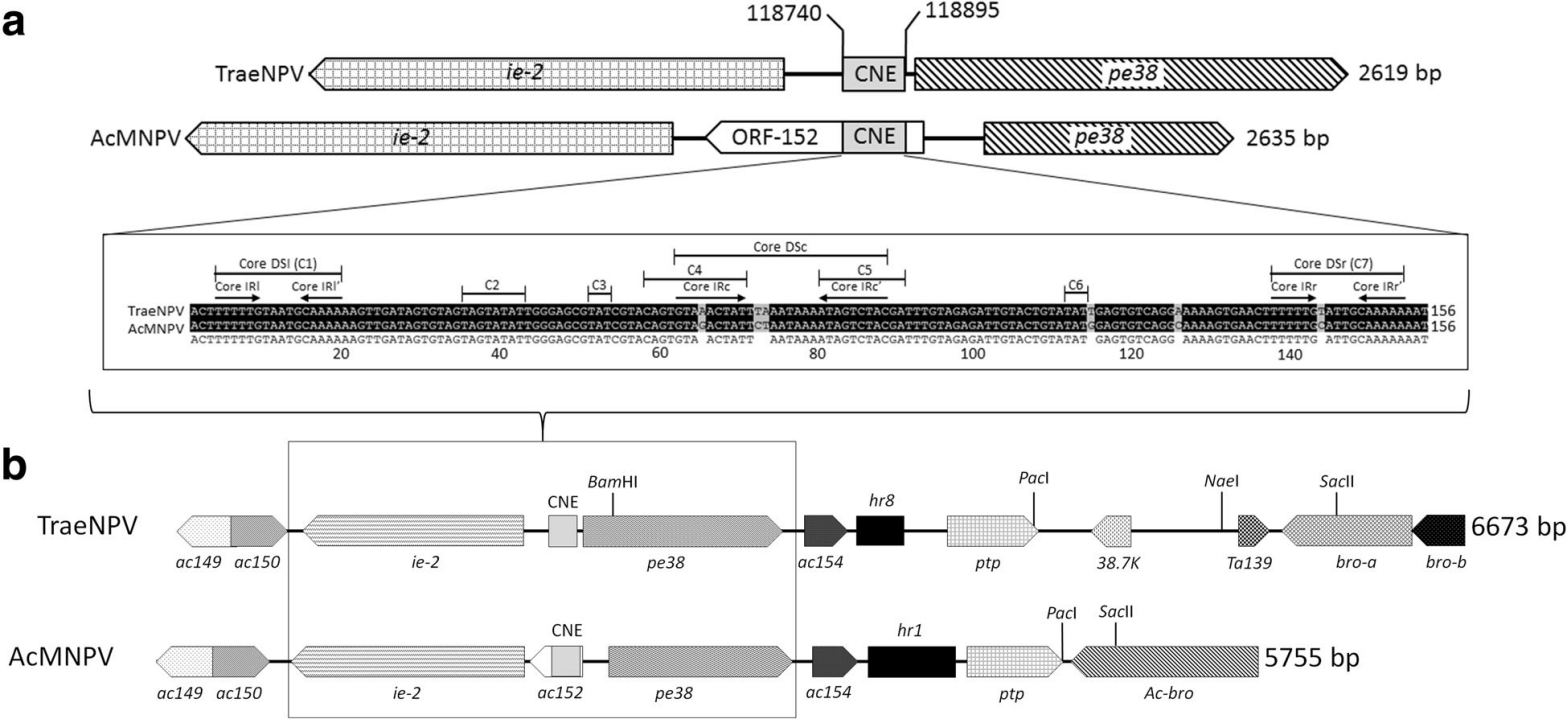
Genomic fragments of TraeNPV and AcMNPV contain the CNE region. (**a**) The location of the CNE for TraeNPV and AcMNPV is flanked by the ie-2 and pe38 genes. The CEN of AcMNPV is overlapped in the ORF-152. The ClustalX alignment of the CNEs of TraeNPV and AcMNPV. The consensus sequence was determined and described by Kikhno [20]. The clusters of conserved nucleotides are indicated (C1~C7). The lines mark the dyad symmetry elements, each of which is indicated by the abbreviation “DS” in conjunction with the lowercase letters (l, c, and r) that specify the DS position in the CNE (left, central, and right, respectively). The inverted repeats are indicated with arrows, and the abbreviation “IR” in conjunction with the letters l, c, and r, assigns each IR pair to a particular DS. (**b**) The comparison of the gene locations by using the relative restriction sites in the TraeNPV with those of the corresponding AcMNPV fragment. Arrows denote ORFs and their direction of transcription. Grey boxes represent the CNE region; black boxes represent the homologous repeat regions (*hr*s). ORF homologues in the corresponding regions are drawn with the same patterns

In addition to these 144 predicted ORFs, other internal spaces were made up of intergenic spaces and common DNA non-coding functional elements (*nfes*), i.e., *homologous region*s (*hr*s). The TraeNPV genome exhibited 8 *hr* (*hr1 ~ 8*) (Fig. 1; Additional file 1: Table S2), and the orientations of the *hrs* were similar to those of AcMNPV. A conserved non-protein-coding genomic element (CNE, 156 bp), which was identified as a member of the *Alphabaculovirus* genus and was speculated to play a role in viral replication, was also found in the TraeNPV genome [20]. The CNE of TraeNPV is located from 118,740 bp to 118,895 bp. For the CNE structures, the seven conserved nucleotide clusters (C1~C7) in the CNE were also found in the CNE of TraeNPV. According to the structure and nucleotide composition, the conserved nucleotide clusters could also be further divided into dyad symmetry elements (DSs) and TAT-containing sequences (Fig. 2a). In the CNE of TraeNPV, three inverted repeats (IR) are presented in the DS left (DSl), DS central (DSc) and DS right (DSr) regions (Fig. 2a). Regarding the orientation of the CNE in TraeNPV, the location of the CNE lacked any ORF overlap in the TraeNPV genome; by contrast, the AcMNPV CNE overlapped with *Ac152* (Fig. 2a). The identity of the CNE showed the highest shared sequence identity (96%) with that of AcMNPV, while the sequence composition of the TraeNPV CNE (AT content 73.8%) revealed a higher AT content than that of AcMNPV (AT content 68.6%).

Based on the experimental data obtained using a CNE-deficient AcMNPV bacmid, the CNE was demonstrated to be a polyfunctional genomic element involved in an essential role in AcMNPV pathogenesis [20]. Moreover, it also demonstrated that the CNE position would not impact the function of the CNE, suggesting that the CNE of TraeNPV might share a similar pathogenesis ability.

### Taxonomic position and phylogenetic analysis of TraeNPV

**Fig. 3:**
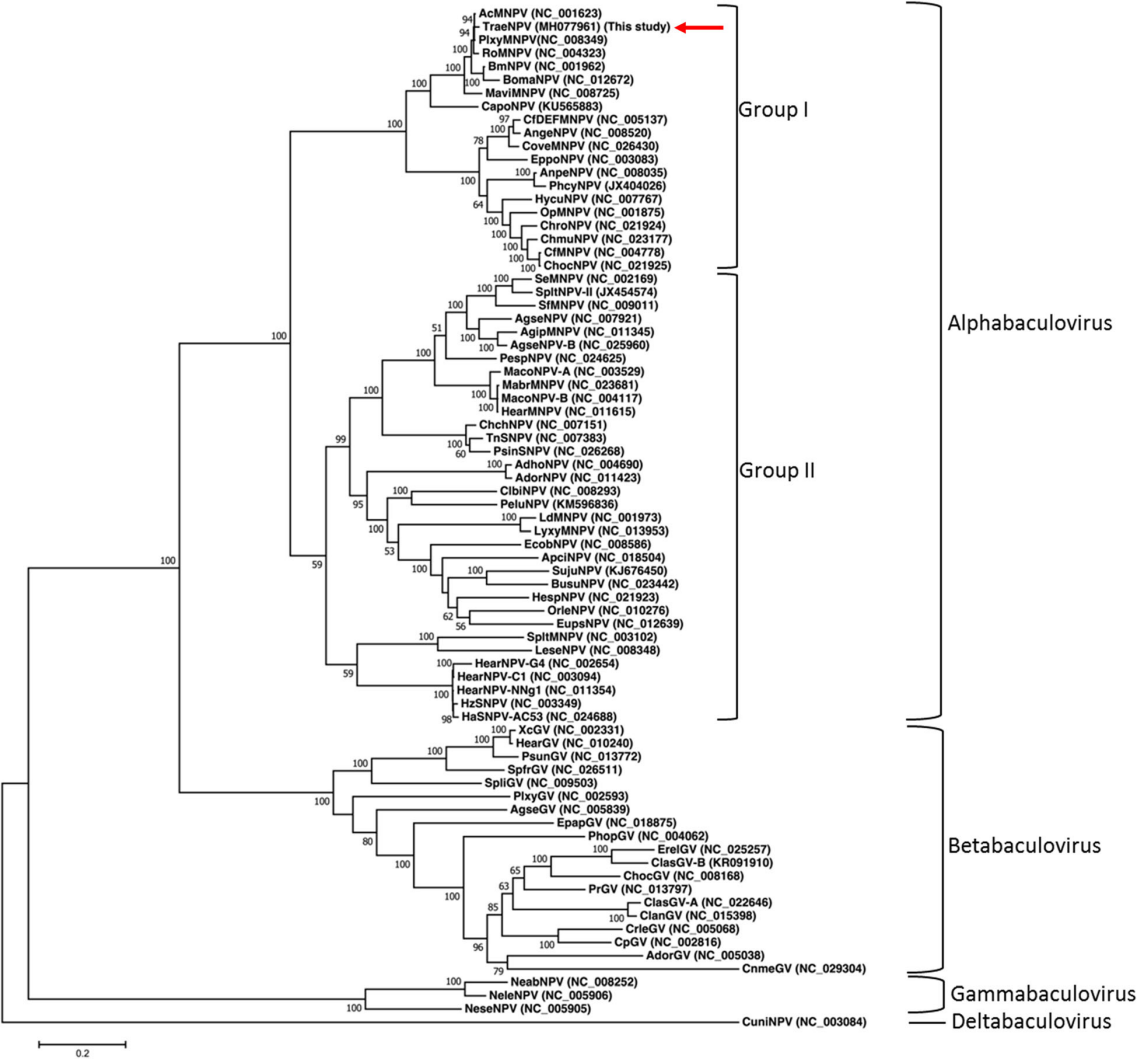
Baculovirus phylogeny inferred from a combined dataset of the 37 baculoviral core protein sequences. An unrooted ML tree is shown. CuniNPV was selected as the outgroup. The numbers at the nodes indicate bootstrap scores above 50% for the ML analyses (100 replicates, ML bootstrap)

The phylogenetic analyses of TraeNPV were performed using NJ and ML methods, and the results were inferred from a data set that combined the amino acid sequences of the 37 baculovirus core genes from 77 whole genomic sequenced baculoviruses (Additional file 1: Table S3) [5, 16]. Both of the phylogenetic trees showed a similar result, and the ML trees revealed higher bootstrap values and are shown in Fig. 3. The family *Baculoviridae* consists of five major clades, the NPVs infecting Lepidoptera (including groups I and II), the GVs, the hymenopteran-specific NPVs, and CuniNPV. This analysis reflected the current systematic assignment of the viruses. Moreover, two subclades within lepidopteran NPV group I resembled the AcMNPV and OpMNPV. The result also indicated that TraeNPV was grouped together with AcMNPV (Fig. 3).

From our previous data, although we attempted to clarify the classification of TraeNPV and its closely related NPVs by K-2-P analysis based on the sequences of *polh*, *lef-8* and *lef-9*, TraeNPV apparently had an ambiguous relationship with its closely related viral species. The results revealed that TraeNPV belonged to the group I baculoviruses and was highly closely related to the BmMNPV and AcMNPV groups [4]. By contrast, the distances for *polh* between TraeNPV and the PlxyNPV, RoNPV, AcMNPV groups exceeded the thresholds of the different viral species, and for all the concatenated *polh*/*lef-8*/*lef-9* sequences, the distances were apparently much greater than the threshold of the same viral isolates; therefore, the limited data indicate an ambiguous situation for TraeNPV [4, 8].

From the comparative genomic studies, the conservation of the general mechanisms underlying baculoviral biology could be speculated; thus, the 37 core genes shared by all the sequenced baculovirus genomes might not only represent the similar function in the mode of viral infection, but they could also reflect the most realistic taxonomic position [20, 21]. Through the whole genome sequencing and the phylogenetic analysis based on 37 baculoviral core genes, it was revealed that TraeNPV is closely related to AcMNPV rather than BmNPV.

### Genome-wide comparisons

**Fig. 4:**
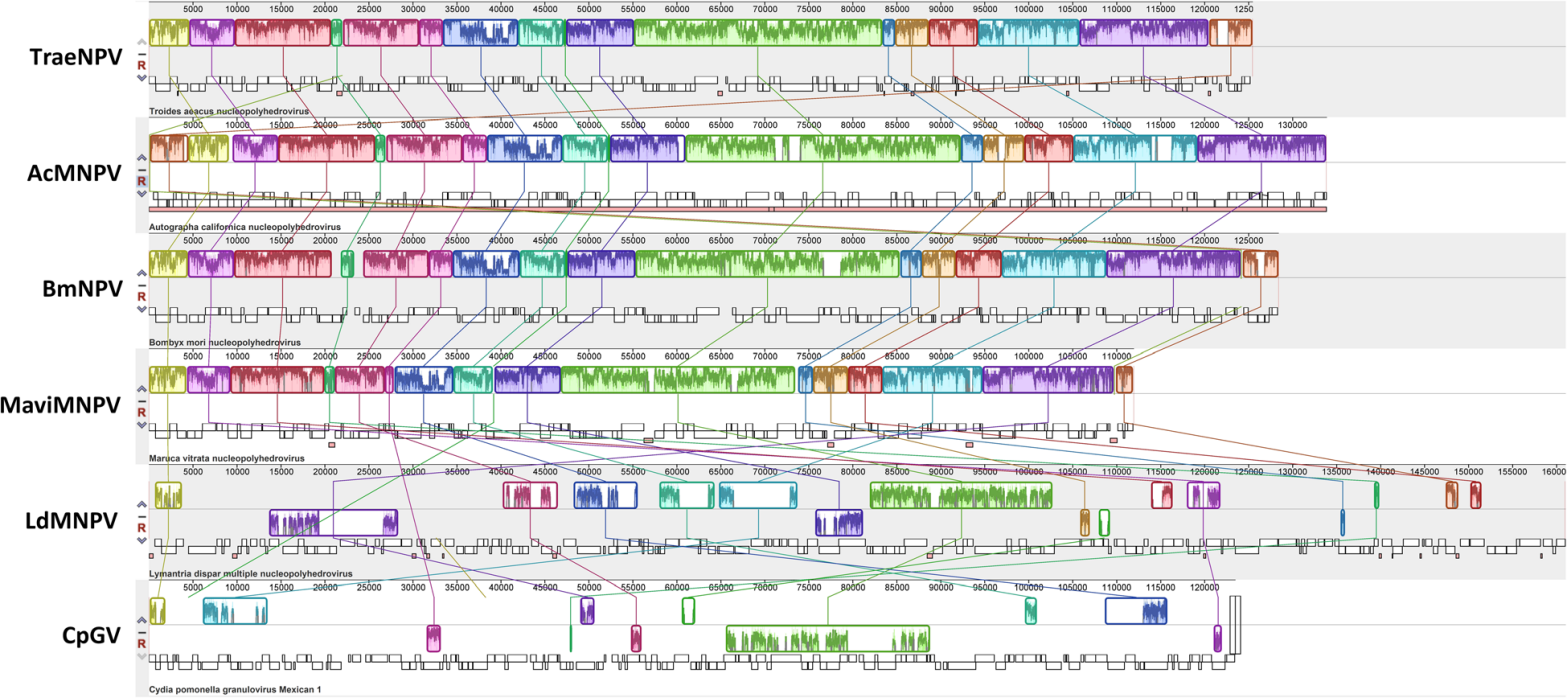
Mauve (multiple alignment of conserved genomic sequence with rearrangements) representation of alphabaculoviruses from group I and II and CpGV. The alignment was performed on collinear sequences in which NPV was a reference sequence and the polh gene was considered as a first ORF (Except AcMNPV). Coloured sections (bordered with a curve that indicates the level of nucleotide similarity) represent the homologous fragments of compared genomes. The section that is located beneath the X-axis shows the inversion of this genome fragment in comparison to the reference

**Fig. 5:**
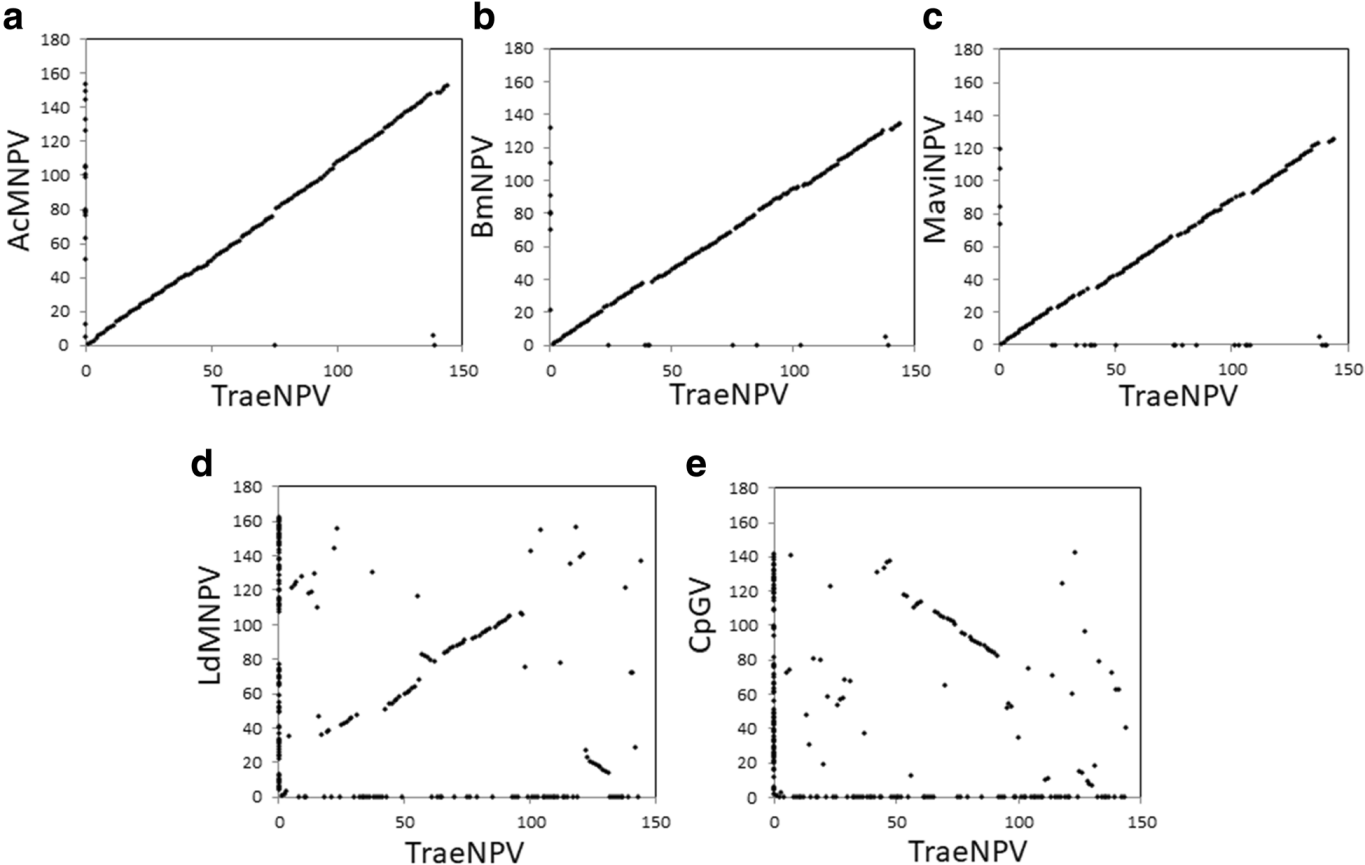
Gene parity plot analysis of TraeNPV in comparison with (**a**) AcMNPV, (**b**) BmNPV, (**c**) MaviNPV, (**d**) LdMNPV and (**e**) CpGV, as indicated. Axes: the relative position of each ORF; dots: ORFs

Comparisons of whole genomes and the gene arrangements of the selected ORFs were performed with CGView, Mauve and a gene parity plot analysis. For the whole genome comparisons, TraeNPV showed a highly similar genomic fragment identity compared to AcMNPV and BmNPV, while a lower shared genomic identity was found between TraeNPV and MaviNPV (Additional file 3: Figure S2). In addition, compared to the TraeNPV genome, there are three locations flanked by the ORFs of *Ta22*/*Ta24*, *Ta74*/*Ta76* and *Ta132*/*Ta141*, which showed a lower shared identity with those of other baculoviruses (Additional file 3: Figure S2). A graphical interpretation of the homologous blocks in viral genomes from alphabaculoviruses from group I and II and from CpGV is shown in Fig. 4. This information also revealed that the conserved segments appeared to be internally free from the genome rearrangement of other baculoviruses; however, a locally collinear block (LCB) deletion between *alk-exo* (*Ta118*) and *p35* (*Ta119*) was found in TraeNPV (Fig. 4). Moreover, the gene arrangement of the TraeNPV genome was highly collinear with that of AcMNPV, BmNPV and MaviNPV. For the gene parity plot analysis, the gene arrangement of the TraeNPV genome showed lower collinearity with LdMNPV and CpGV and the ORFs displayed a much more dispersed pattern (Fig. 5).

A further comparison of the genomic fragments from *Ta132* to *Ta141* to that of AcMNPV revealed a 1576 bp DNA fragment insertion from the nucleotide positions 121,403 bp to 122,979 bp in the TraNPV genome (Fig. 2b). Within the inserted DNA fragment, one novel gene (*Ta139*) and one duplicate gene were found; in addition, the restriction enzyme profile also revealed a difference in the *Ta132*/*Ta141* fragment relative to that of AcMNPV (Fig. 2b). Although TraeNPV was similar to AcMNPV and BmNPV in terms of gene organization, the presence of a different region was found upon genome-wide analysis.

According to the comparative analysis of baculoviral genomes, baculoviruses are highly diverse in terms of their GC content, genome length, gene content, and gene organization. These characteristics could reflect the evolutionary history of baculoviruses in adapting to different hosts [21, 22]. Based on the gene content (two novel ORFs were found in TraeNPV and were lacking 15 AcMNPV homologous ORFs) and genomic length (shorter than AcMNPV), TraeNPV might be distinct from AcMNPV.

### Comparison of TraeNPV ORFs with other baculoviruses

**Fig. 6:**
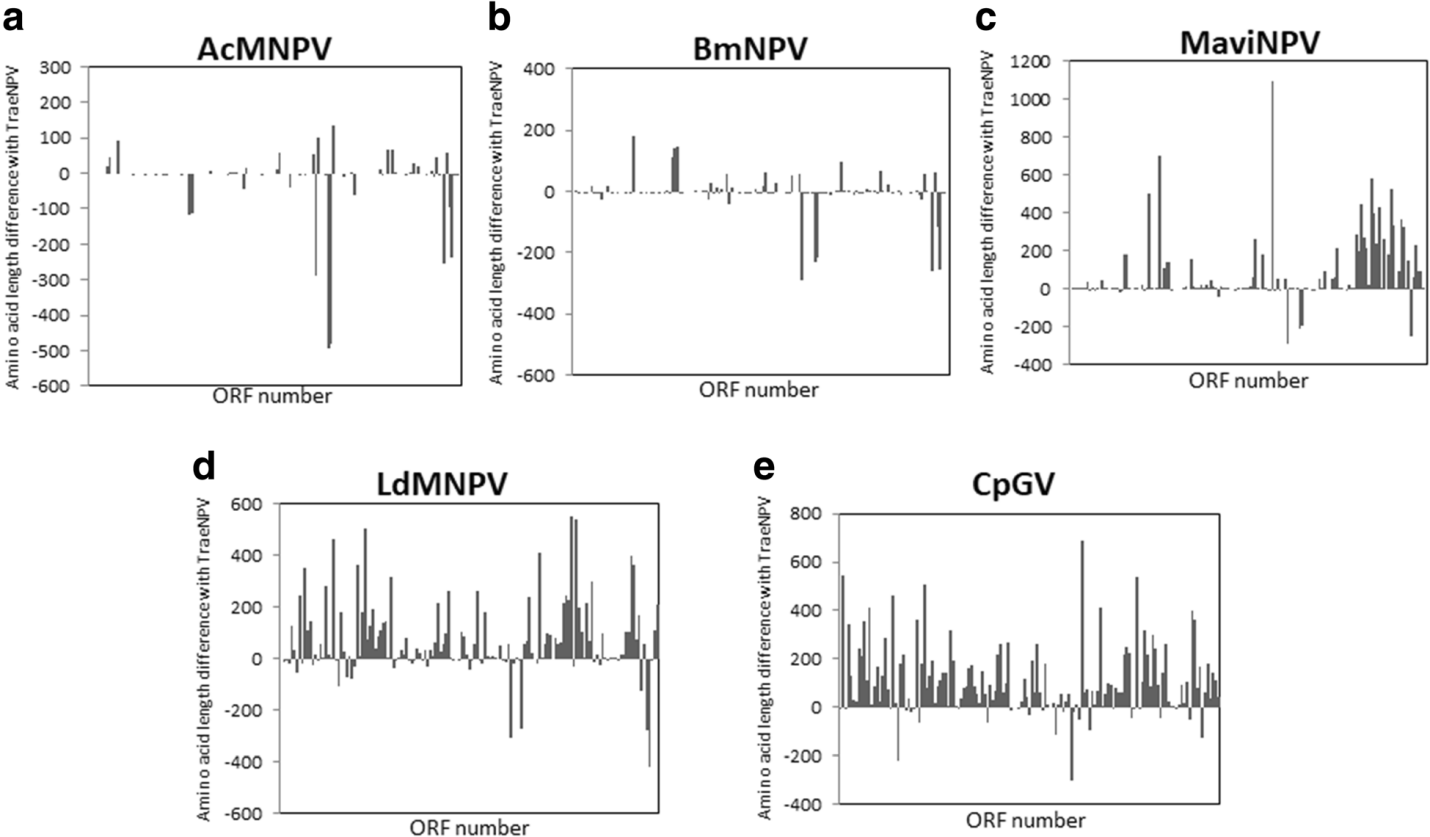
Amino acid length difference for TraeNPV compared to (**a**) AcMNPV, (**b**) BmNPV, (**c**) MaviNPV, (**d**) LdMNPV and (**e**) CpGV, as indicated. X-axis: the relative position of each ORF; Y-axis dots: amino acid differences

TraeNPV shares 142 ORFs with AcMNPV, 136 ORFs with BmNPV, 124 ORFs with MaviMNPV, 90 with LdMNPV and 74 with CpGV. The average shared amino acid sequence identity between TraeNPV and AcMNPV, BmNPV, MaviMNPV, LdMNPV and CpGV were 90.96, 86.61, 78.71, 33.20, and 25.61%, respectively. Based on the presented data, TraeNPV is closely related to AcMNPV; of the 142 ORFs that are common to TraeNPV and AcMNPV, only 2 ORFs sharing 100% identity and 97 ORFs sharing > 95% identity were found. Of the other 43 ORFs, 18 ORFs sharing 95–90% identity, 12 ORFs sharing 89–80% identity and 13 ORFs sharing < 80% identity were found. It is notable that there were three ORFs, *Ta95* (*Ac106–107*), *Ta103* (*Ac118*) and *Ta126* (*odv-e18*), that had low shared identities (39, 52 and 61%, respectively) compared to those of the AcMNPV homologues due to the variations in the amino acid lengths, suggesting that there might be amino acid variations between TraeNPV and AcMNPV. In fact, the further analysis showed that variations were found in the amino acid lengths and identities between TraeNPV, AcMNPV and BmNPV (Figs. 1 and 6; Additional file 1: Table S2). In addition, it also showed clear amino acid length differences compared to those of MaviMNPV, LdMNPV and CpGV.

TraeNPV lacks 15 ORFs in AcMNPV and 7 ORFs in BmNPV (Table 1). In addition, there are two pairs of adjacent AcMNPV ORFs (*Ac58*/*Ac59* and *Ac106*/*Ac107*) that were fused together into single ORFs (*Ta51* and *Ta95*, respectively) in TraeNPV. As reported for *Rachiplusia ou* MNPV-R1, the re-sequencing of these regions in AcMNPV-C6 indicated that the ORF pairs occurred as a single ORF in the AcMNPV-C6 stock [23]. Homologues of these ORFs were also found in other baculovirus genomes in which they were fused into a single ORF (Additional file 1: Table S2).

**Table 1:** AcMNPV and BmNPV ORFs with no homologues in the TraeNPV genome

AcMNPV			BmNPV		
^a^ORF	Length (aa)	Name	^a^ORF	Length (aa)	Name
3	53	*ctx*	22	317	*bro-a*
7	201	*orf603*	70 (*Ac87*)	126	*vp15*
12	217		80	239	*bro-b*
20	69		81	318	*bro-c*
58	57		91 (*Ac108*)	105	
70	290	*hcf-1*	111 (*Ac134*)	70	
84	188		132	241	*bro-e*
85	53				
86	694	*PNK/PNL*			
87 (*Bm70*)	126	*vp15*			
108 (*Bm91*)	105				
112–113	256	*apsup*			
134 (*Bm111*)	803				
140	60				
152	92				

^a^ORFs in the brackets are homologues ORFs between AcMNPV and BmNPV

### TraeNPV structural genes

TraeNPV contains 35 baculovirus structural genes, which were listed by Hayakawa et al. (2000), Jehle et al. (2006) and Thumbi et al. (2013) [5, 21, 24], and only the p15 (Ac87) gene was absent from the TraeNPV genome (Table 2). Of the 35 structural proteins, the P74 protein is associated with occluded virions and is required for oral infectivity [25, 26]; the VP1054 protein is required for AcMNPV nucleocapsid formation [27]; the P10 protein has been shown to be involved in the formation and stability of polyhedra and may influence cell lysis late in infection [28–30]; VP80 is associated with both ODV and BV in AcMNPV and OpMNPV [31, 32]; and ORF1629 is associated with the basal end of nucleocapsids and is essential for AcMNPV viability [33, 34]. The GP64 protein is the envelope fusion protein of the budded virus, and it is specific to the group I NPVs [35, 36]. Another envelope fusion protein that is functionally analogous to the GP64 protein called Ld130 is present in all lepidopteran and dipteran baculoviruses that have been completely sequenced, including those that contain *gp64*. The TraeNPV genome also contains these proteins, and it encodes both GP64 (*Ta113*) and Ld130 (*Ta14*). It has been suggested that Ld130 homologues may play a role in the ancient envelope fusion protein, and its fusion function was substituted by *gp64*; the co-existence of this gene with *gp64* might occur because it has other essential functions [36]. There are several genes that encode capsid-associated proteins (*vp39* and *vp91*), ODV envelope proteins (*odv-e18, −e25*, −*e56,* and -*e66*), DNA binding protein (*p6.9*)*,* and the tegument protein (*gp41*) that is also associated with BV production [37, 38]. Most of these structural genes have highly shared identities in AcMNPV, specifically > 95% shared identities, suggesting that the structure of the TraeNPV may be similar to that of AcMNPV. The structural protein of TraeNPV shared a high similarity with that of AcMNPV, and there are four structural genes with slightly lower shared identifies with AcMNPV, namely, *polh* (*Ta1*; 88%), *gp64* (*Ta113*; 92%), *odv-e18* (*Ta126*; 61%) and *odv-e26* (*Ta8*; 89%) (Additional file 1: Table S2). It has been reported that the AcMNPV *polh* consists of a mosaic of group I and group II NPV-specific sequences and it has a chimerical structure [39]. Interestingly, a low shared identity (88%) for *polh* was found between TraeNPV and AcMNPV, suggesting that this difference may be related to a process in baculovirus evolution.

**Table 2:** Baculovirus gene category in TraeNPV

Category	Genes in TraeNPV						
Replication genes (19)	*38.7 K* (*Ta5*)	*lef-1*	*dbp1*	*gta*	*pcna-a*	*pcna-b*	*DNA-pol*
	*lef-3*	*helicase*	*p35*	*me53*	*ie-0*	*ie-1*	*ie-2*
	*38.7 K* (*Ta138*)	*lef-2*	*pe38* ^a^	*lef-7* ^a^	*39K* ^a^		
Transcription genes (13)	*lef-6*	*lef-11*	*p47*	*lef-12*	*lef-8*	*lef-10*	*lef-9*
	*vlf-1*	*lef-4*	*lef-5*	*lef-7* ^a^	*pe38* ^a^	*39K* ^a^	
Structural genes (35)	*polh*	*orf1629*	*pif-2*	*efp/ld130*	*odv-e66*	*ac53*	*vp1054*
	*fp25K*	*desmop*	*ac68*	*ac78*	*gp41*	*ac81*	*vp91/p95*
	*vp39*	*p33*	*odv-e25*	*p6.9*	*p40*	*vp80*	*pif-3*
	*pif-1*	*gp64*	*p24*	*gp16*	*pp34*	*p10*	*p74*
	*p49*	*odv-e18*	*odv-e27*	*odv-e56*	*ac109*	*ac16*	*19kda*
Auxiliary genes (14)	*ptp*	*egt*	*alk-exo*	*v-cath*	*arif-1*	*sod*	*fgf*
	*ubiquitin*	*pkip*	*p26*	*tlp20*	*p12*	*cg30*	*chitinase*

Genes are categorized based on their functions during virus replication

^a^Gene, which is involved in multiple funcitons

### Transcription-specific genes

A total of 13 genes involved in late baculovirus gene transcription that are all present on other baculovirus genomes [5, 21, 24] are also present in the TraeNPV genome, including *lef 4–12*, *39 K*, *p47, vlf-1* and *pe38* (Table 2). Of these genes, 10 genes (*lef-4~ − 6, − 8~ − 12, 39 k,* and *p47*) are required for optimal levels of late gene transcription in the AcMNPV genome [40, 41]. These 10 proteins play a role in the virus-encoded RNA polymerase that recognizes a late promoter element, RTAAG (R = A, T, or G) [42]. Moreover, *lef-4, lef-8, lef-9,* and *p47* form a minimal complex with late polymerase activity [43]. Additionally, a conserved gene, *vlf-1 might*, regulates very late gene transcription and may be involved in DNA processing [44–46]. These genes had high shared identities with AcMNPV, at 84–98%, suggesting that a similar mechanism for late gene transcription might occur in the Baculoviridae group.

### DNA replication genes

A major group of conserved genes involved in DNA replication was described previously [5, 21, 24, 47]. AcMNPV and OpMNPV contain 5 genes that are essential for transient DNA replication (*ie-1, lef-1*, *lef-2, lef-3* and *helicase*) and 5 non-essential ones that stimulate transient DNA replication genes (*dna-pol, p35, ie-2, lef-7,* and *pe38*) [48–50]. These 10 genes are all present in the TraeNPV genome (Table 2). Six of these 10 genes (*ie-1, lef-1*, *lef-2, lef-3*, *helicase* and *dna-pol)* have previously been reported as essential DNA replication factors for baculoviruses, indicating that baculoviruses share a common DNA replication mechanism [50].

**Fig. 7:**
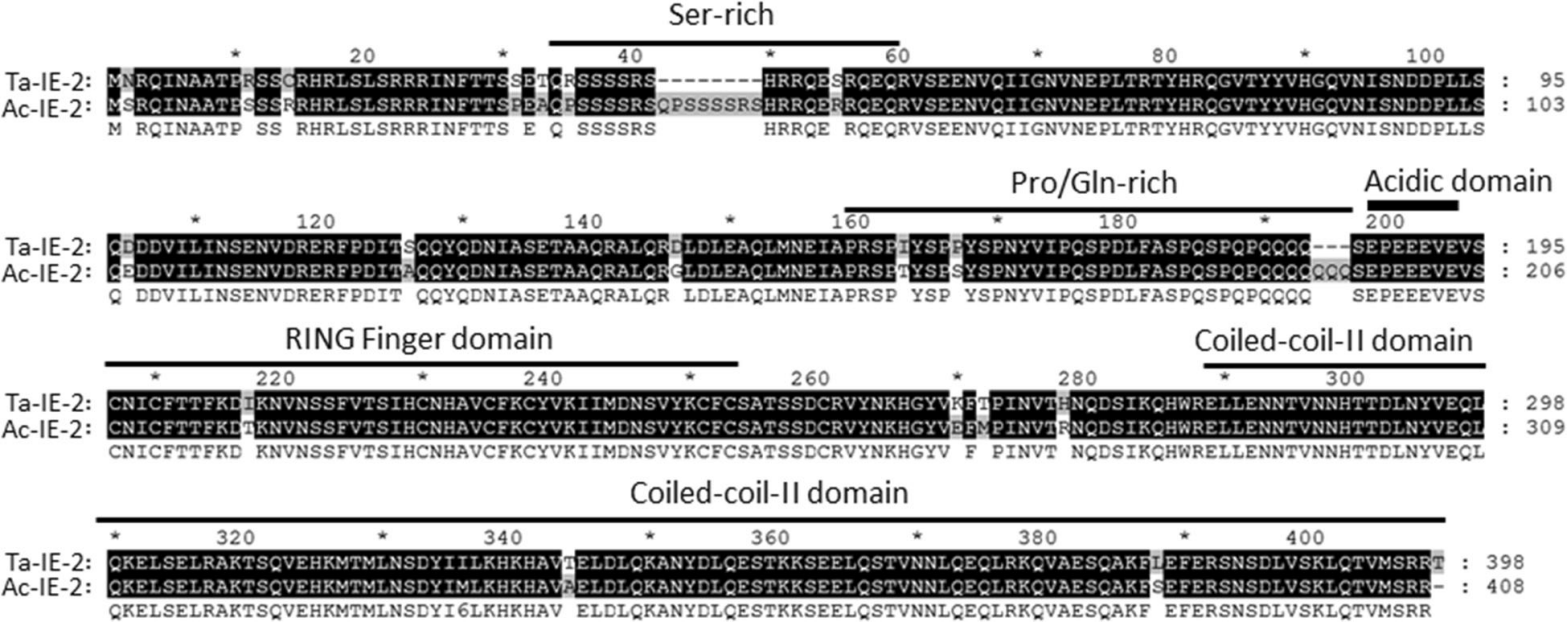
Alignment of IE-2 amino acid sequences. The identical residues occupying > 50% of the aligned positions are shaded in black, and residues similar to the conserved residues or to one another are shaded in grey. The lines above the aligned sequences indicate the locations of different functional motifs. The acidic domain required for transcriptional activation is indicated with a thick line

The other DNA replication genes, such as single-stranded DNA-binding protein (*dbp1*) and immediate-early gene (*me53*), which have been implicated in DNA replication, were also found in TraeNPV (Table 2) [51]. During viral infections, the host cell RNA polymerase II is often transactivated by genes such as *ie-0*, *ie-1*, *ie-2* and *pe38*. These genes are conserved relative to those of AcMNPV (84–98%); however, a small variant form of the IE-2 protein was found between TraeNPV and other closely related NPVs (Fig. 7). Although the TraeNPV IE-2 amino acid sequence shared 92% identity with that of the AcMNPV IE-2, the serine-rich and proline/glutamine-rich domains involved in activating a subset of early baculovirus promoters by AcMNPV IE-2 have a short deletion in the TraeNPV sequence (Fig. 7) [52]. A RING finger domain, which is required for cell cycle arrest, E3 ubiquitin ligase activity, and nuclear focus association; and a predicted coiled-coil region (coiled-coil-II), which is involved in self-interaction and association with nuclear foci, were strongly conserved in TraeNPV IE-2 and AcMNPV [53–56].

The TraeNPV genome encodes two PCNA proteins (*Ta40* and *Ta41*), and both proteins had low shared amino acid identities with AcMNPV (53 and 36%). Further investigation revealed that a single DNA base deletion resulted in two truncated forms of PCNA proteins, while a proliferating cell nuclear antigen PCNA protein may be involved in viral DNA replication, DNA recombination or DNA repair, but not the essential function of DNA replication, suggesting that the side effect of DNA replication may present differences between different viral species and hosts [57, 58].

### Genes with auxiliary functions

Auxiliary genes are not essential for viral replication, but they provide a selective advantage for increasing the virus production/survival at either the cellular or organismal level [21]. A total of eighteen auxiliary genes have homologues in TraeNPV (Table 2). These auxiliary genes in TraeNPV were 90–100% identical in terms of amino acid sequences compared to those of AcMNPV, except for *alk-exo* and *arif-1*. The TraeNPV *alk-exo* was 81% identical to AcMNPV and its *arif-1* was 72% identical to that of AcMNPV. According to the analysis, the lower shared identities were caused by amino acid length variations. The a*rif*-*1,* which is involved in the sequential rearrangement of the actin cytoskeleton, is found only in NPVs [59]. Therefore, it may contribute to the morphological differences between different NPV and GV-infected cells.

### Homologous regions (*hrs*)

Homologous regions (*hrs*) is one of the feature found in most baculovirus genomes and locate in multiple sites in the genomes [60]. The structure of each *hr* contains a palindrome, which is flanked by direct repeats. *Hrs* function as origins of NPVs and GVs replication [61] and also serve as RNA polymerase II-mediated transcription enhancers in early baculovirus promoters in NPVs [62]. Recently, it has been reported that no single homologous repeat region is essential for the DNA replication of AcMNPV [63].

**Fig. 8:**
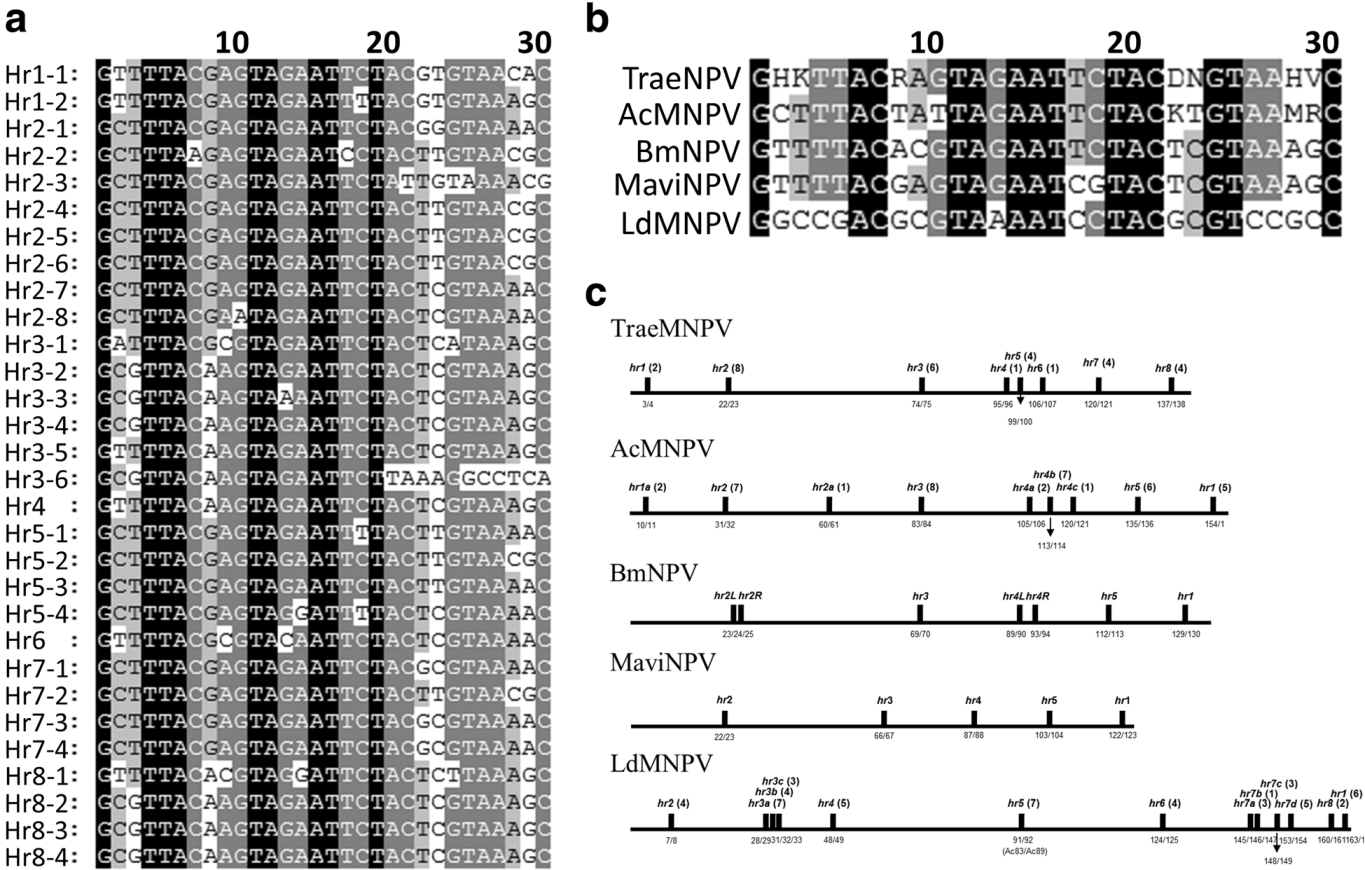
Comparison of TraeNPV *hr*. palindromes with (**a**) each hr. palindrome, which was identified from the TraeNPV genome; and (**b**) palindrome consensus sequences from other baculoviruses. The alignment of the consensus hr. palindrome from TraeNPV, AcMNPV, BmNPV, MaviNPV and LdMNPV; and (**c**) a comparison of the genomic context of the hrs and hr. locations relative to the homologous ORFs between TraeNPV, AcMNPV, BmNPV, MaviNPV and LdMNPV in the linearized genomes. The ORFs flanking the hrs: below the line. Grey rectangles: the major inserts relative to AcMNPV and the ORFs within the inserts are shown above the line. For consistency, all the linearized genomes start with polh, but the hrs and ORF numbers remain the same as in the original papers

The TraeNPV genome contained eight homologous repeat regions (*hr1*, *hr2*, *hr3*, *hr4*, *hr5*, *hr6*, *hr7* and *hr8*) that included one to eight palindrome repeats for a total of 30 repeats (Fig. 8a and c) and accounted for 0.72% of the genome. Similar to the AcMNPV palindrome sequence [9], the TraeNPV *hr* palindrome consensus GHKTTACRAGTAGAATTCTACDNGTAAHVC shows a 23/30 matched palindrome (Fig. 8b) and the palindromic consensus sequence included seven highly variable positions (Fig. 8b). All the nucleotides in the palindrome were conserved, except for the twenty-second nucleotide. In addition, the LdMNPV consensus *hr* palindrome shared 43.3% of its sequence identity with the TraeNPV consensus *hr* sequence (Fig. 8b). The genomic positions of the TraeNPV regions *hr1* - *hr8* were conserved with the genomic positions of AcMNPV [9]; however, a lack of AcMNPV *hr2-a* was found in the TraeNPV genome (Fig. 8c).

### Baculovirus repeated ORFs (*bro* genes)

A striking feature of most lepidopteran and dipteran NPVs sequenced to date and in some of the GVs is the presence of one to 16 copies of *bro* genes. Typically, *bro* genes are highly conserved, repetitive and widely distributed amongst insect DNA viruses [64]. The function of these genes is unclear, but they have been shown to bind to DNA [65]. These genes have also been found to be associated with the viral genome rearrangement regions [66]. During the process of baculovirus replication, the viruses that synthesize mRNAs in the nucleus and this mRNA should be exported to the cytoplasm, while some viral proteins produced in the cytoplasm must be imported into the nucleus. It was demonstrated that the BRO proteins of BmNPV play a role in the function of the nucleocytoplasmic shuttling proteins that utilize the CRM1-mediated nuclear export pathway [67].

TraeNPV contained two *bro* genes, which were named *bro-a* and *bro-b* based on their order in the genome (Fig. 1; Additional file 1: Table S2). Most BROs contained a core sequence of 41 aa at the N-terminal half and several different domains throughout the sequence. The *bro* gene family has been divided into four groups based on the similarity of those domains [12]. Both of the TraeNPV bro genes, namely *Ta-bro-a* (*Ta141*) and *-b (Ta142*) (which were homologues of *Bm-bro-d*) belong to group III. Moreover, two TraeNPV *bro* genes encoded small fragments of truncated protein (234 aa and 92 aa). It has been reported that mutations in the leucine-rich region of Bm-BRO proteins resulted in the nuclear accumulation of transiently expressed proteins; however, the mutant Bm-BRO-D with an altered nuclear export signal (NES) did not show nuclear accumulation in the infected cells due to a reduction in RNA synthesis [67], suggesting that the truncated BRO protein in TraeNPV may share a similar function as that of Bm-BRO-D.

### Genes involved in host range determination

Baculoviruses usually showed high specificity to a few, or even individual, insect species [68, 69]. For this reason, a variety of efforts has been made to understand the baculoviral genes that are related to their host range. Many viruses encode a variety of proteins related to the host range; AcMNPV is the most widely researched member of the *Baculoviridae*. AcMNPV contains several genes that involved in host range determination, including *p143 (helicase)*, *hrf-1* (*host range factor 1*), *hcf-1* (*host cell-specific factor 1*), *ie-2* and *p35* [69–72]. Of these genes, *p35* and *iap* (inhibitor of apoptosis), are two major families of anti-apoptosis genes, which are commonly found in baculovirus genomes [73, 74].

The inhibition of vary caspase pathways by the *p35* and its homologue *p49* have been demonstracted [75]. The *p35* and *p49* are found in few sequenced baculoviruses, such as AcMNPV and *Spodoptera litura* MNPV (SpltMNPV) [9, 76]. For another anti-apoptosis gene family, the anti-apoptotic inhibition of IAP proteins has been demonstrated either directly or indirectly during baculovirus infection in permissive cells or heterogeneous insect cells in AcMNPV, *Anticarsia gemmatalis* MNPV (AgMNPV), *Cydia pomonella* granulovirus (CpGV), *Epiphyas postvittana* NPV (EppoNPV), *Helicoverpa armigera* NPV (HearNPV), *Hyphantria cunea* NPV (HycuNPV), *Leucania separata* MNPV (LeseMNPV), *Orgyia pseudotsugata* MNPV (OpMNPV), *S. littoralis* NPV (SpliNPV) and LyxyMNPV [75, 77–88]. Similar to AcMNPV, in the TraeNPV genome, *p35* (*Ta119*) and two *iap*s, *iap1* (*Ta18*) and *iap2* (*Ta62*), were identified. The amino acid identities of these three proteins are 97, 95 and 84% shared with those of AcMNPV; it is speculated that these proteins might share similar activities in the host cells.

Recently, *ld-apsup* (*ld109*), a novel gene that inhibits apoptosis in LdMNPV-infected Ld652Y cells, was identified and its anti-apoptotic activities and mechanism were demonstrated [89, 90]. According to a survey of the genome data, AcMNPV (*Ac112–113*) and other 17 baculoviruses contained *apsup* homologue genes in their genomes [89]. Interestingly, a lack of *Ac112–113* was found in the TraeNPV genome (Table 1), and more extensive experiments may be performed to investigate the host range issue.

### TraeNPV truncated and duplicate genes

There were three truncated ORFs (*pcna-a*/*pcna-b*, *he65-a*/*he65-b* and *bro-a*/*bro-b*) and one duplicated ORF (*38.7 K* in the *Ta5* and *Ta138* locations) located in the TraeNPV genome. All of the truncated ORFs showed low shared identities with their homologues in AcMNPV. For *pcna-a*/*pcna-b* (*Ta40/Ta41*), the amino acid identities are 53 and 36% shared, respectively, compared to *Ac49*; 4 and 12% in *he65-a*/*he65-b* (*Ta93/Ta94*) compared to *Ac105*; and 56 and 16% in *bro-a*/*bro-b* (*Ta140/Ta141*) compared to *Ac2*. For these truncated genes, the nucleotide deletions leading to the introduction of stop codons were found in both *pcna-a*/*pcna-b* (*Ta40/Ta41*) and *bro-a*/*bro-b* (*Ta140/Ta141*). For *pcna-a*/*pcna-b* (*Ta40/Ta41*), a one-bp deletion was found in the downstream 398 bp (+ 398 bp) of *ac-pcna*; this deletion resulted in the introduction of a stop codon (TGA) in the + 434 bp, and thus a second *pcna-b* start codon was found between + 436 bp and the end of this gene. In *bro-a*/*bro-b* (*Ta140/Ta141*)*,* a seven-bp deletion was found 222 bp downstream (+ 222 bp) of *ac-bro*, and this deletion resulted in the introduction of a stop codon (TGA) in the − 284 bp. Thus, a second *bro-b* start codon was found between + 283 bp and the end of this gene. For *he65-a*/*he65-b* (*Ta93/Ta94*), instead of the full-length *he65* (553 aa) in AcMNPV, TraeNPV encoded two smaller proteins, *he65-a* (58 aa) and *he65-b* (72 aa). The HE65 protein is one of the RNA ligase families, and it acts as an early transcription gene involved in RNA replication, transcription and modification as well as in G-actin localization in the nucleus during AcMNPV cell infection. Although truncated he65 was found in the genome, it is considered a nonessential protein for AcMNPV and BmNPV [91, 92].

One pair of genes (*Ta5*/*Ta138*) was identified as duplicated homologues of *38.7 K* in the TraeNPV genome. This duplicate gene (*Ta138*) showed low shared identities with the homologues of AcMNPV (15%).

### Unique TraeNPV ORFs

Two genes are unique in the TraeNPV genome, including *Ta75* and *Ta139* (Fig. 1; Additional file 1: Table S2). These unique ORFs were small in size (55–60 aa). Both *Ta75* and *Ta139* had no any baculovirus homologue and no significant BLAST database hit. However, the promoter region should be predicted in the future to evaluate the transcriptional contribution to TraeNPV.

### Comparison of TraeNPV to AcMNPV

Based on the sequence analysis, TraeNPV was highly similar to AcMNPV. The phylogenetic analysis indicated that TraeNPV belonged to *Alphabaculovirus* Group I. However, there were still some distinctions in the genomic features and gene content between these two viruses. The most significant difference between TraeMNPV and AcMNPV was that the TraeNPV genome is 8417 bp smaller than the AcMNPV genome (133,894 bp) and it contains 15 fewer ORFs (Table 1), while the TraeNPV genome contained two ORFs that were not found in the AcMNPV genome (Additional file 1: Table S2). Moreover, according to the data on the in silico restriction enzyme fragment length polymorphism (in silico RFLP) pattern using *Bam*HI, TraeNPV showed a different pattern compared to that of AcMNPV (Additional file 4: Figure S3). The AcMNPV genome contains 15 ORFs, which were not found in the TraeNPV genome. Two genes that encode HCF-1 and APSUP were described as the host range determination factors in baculoviruses [89, 90]. It has been demonstrated that the AcMNPV HCF-1 protein is an essential viral factor for the productive NPV infection of TN-368 cells [93, 94]. Recently, a novel anti-apoptotic protein, APSUP, was identified in LdMNPV [95]; moreover, it has been demonstrated that the full-length Ld-Apsup could work against the apoptosis of Ld652Y cells induced by exposure to actinomycin D and UV and could interact with Ld-Dronc to prevent cells from undergoing apoptosis. The baculovirus host range likely involves a complicated array of viral and cellular factors. Based on the data from the genomic analysis, a lack of *Ac112–113* was found in the TraeNPV genome (Table 1), and more extensive experiments may be performed to uncover more evidence regarding the host range issue.

There were 142 ORFs in common between TraeNPV and AcMNPV, and their order is mostly identical. However, several of these ORFs had different lengths, as shown in Fig. 6. These genes included *arif-1*, *IAP2*, *vp91/p95*, *pp34*, *alk-exo*, *odv-e18* and *ie-2* as well as other genes with unassigned functions. Moreover, three pairs of truncated genes were found in the TraeNPV genome, namely, *pcna-a*/*pcna-b*, *he65-a*/*he65-b* and *bro-a*/*bro-b.* These truncated genes also showed amino acid length variations between TraeNPV and AcMNPV (Fig. 6). The *hrs* of TraeNPV are similar to those of AcMNPV in terms of their position, numbers and orientations, while there was no *hr2a* in TraeNPV. The gene content, ORF length and *hr* are possible candidates for regulators of the different virulence levels between two closely relative species [67], which might be the case for TraeNPV and AcMNPV.

## Conclusions

In conclusion, TraeNPV showed a high degree of collinearity and shared sequence identity with AcMNPV. However, these two viruses showed different host ranges and geographical distribution. To date, TraeNPV has only been isolated from *T. aeacus*, which is a native butterfly species under conservation in Taiwan. Furthermore, although the genome sequence analysis revealed that TraeNPV lacks 15 homologous genes from AcMNPV, TraeNPV gained two novel unique genes. Interestingly, there were two host range determination genes, *hcf-1* and *apsup*, in AcMNPV (and also in other alphabaculoviruses) that were not found in TraeNPV. These findings were very interesting and worthy of further studies to collect more evidence about the host range issue. Based on our analytical data, TraeNPV would be clarified as a new NPV species, which has defective AcMNPV genomic features. The lack of *hcf-1* and *apsup* in the genomic sequence data for TraeNPV could provide useful information for understanding the baculoviral host ranges and for gaining evolutionary insights.

## Methods

### Viral DNA extraction and DNA sequencing

Diseased *T. aeacus* larvae samples were homogenized in 1.7 mL microcentrifuge tubes and then examined under a light microscope for viral occlusion bodies (OBs). To obtain the OBs, the samples were centrifuged at 14,000×*g* at 4 °C for 10 min and the supernatants were removed. The pellets were washed in 1 *×* TE buffer (10 mM Tris-HCl, and 1 mM EDTA, pH 7.6) and centrifuged three times at 14,000×*g* at 4 °C for 10 min. The pellets were then resuspended in 1 *×* TE buffer with a final concentration of 1% (w/v) SDS and then incubated with proteinase K (0.25 mg/ml) at 56 °C for 3 h. The total DNA (including the host and viral DNA) was extracted using previously published methods [96]. A sequencing library was prepared following the standard protocol from the NEBNext Ultra II DNA Library Prep Kit for Illumina (NEB) and sequenced with an Illumina MiSeq sequencer with paired-end (PE) technology for 2 × 300 bp.

### Data pre-processing and bioinformatics analysis

The total PE reads were conducted for sequencing adapter identification and then trimmed by cutadapt [97]. Ambiguous bases and bases with lower quality values were removed by PRINseq [98] from either the 5′- or 3′-end. The final high quality reads were selected using NGS QC Toolkit [99] with the default parameters (Additional file 1: Table S4). These trimmed reads were then subjected to genome assembly and annotation by bioinformatics analysis (Additional file 5: Figure S4).

The strategy for TraeNPV genome assembly is to employ the longer paired-end (PE) reads. The genome assembly approach used in this study is reference-guided assembly, with benefits from the reference organism. The reference species is identified as the top-ranked individual with the highest read count by mapping PE reads against the collection of viral genomes from NCBI GenBank. MIRA [100], one of the reference-guide assembly types, maps sequencing reads against reference species to generate the genome sequence of target species. Gap elimination was applied using an in-house script programme by mapping the quality PE reads and contigs iteratively until convergence was reached. Contigs are the joined paired-end reads found by using COPE [101] and assembled contigs were found by de novo assembly, with SOAPdenovo [102]. Draft genome gap filling and gene coding region validation were performed by Sanger sequencing to complete the final genome and gene annotation, respectively. The designed primer sets for PCR validation are listed in Additional file 1: Tables S5 and S6.

The genome annotations were performed with both NCBI ORF finder (https://www.ncbi.nlm.nih.gov/orffinder/) and Glimmer [103] to identify the open reading frames in the genome. Repetitive sequence regions were detected by RepeatMasker (http://www.repeatmasker.org/). CD-HIT and BLASTN in the NCBI BLAST package were used to identify the correctness of the predicted genes and the corresponding sequence identities. A circular map of the viral genome was generated by CGView [104].

### Phylogenetic analysis

The phylogenetic tree was inferred from a data set of concatenated amino acid sequences from the 37 baculovirus core genes [5, 16] of the 77 baculoviruses that were completely sequenced at the time of analysis (Additional file 1: Table S3). A maximum likelihood (ML) analysis was performed using *MEGA* version 7.0 [105]. *Culex nigripalpus* NPV (CuniNPV) [106] was selected as the out-group. A bootstrap analysis was performed to evaluate the robustness of the phylogenies using 100 replicates for ML analysis.

### Comparative genomic analysis

Both the whole genome and all the putative ORFs of TraeNPV were subjected to a comparative genomic analysis with 4 alphabaculoviruses (3 group I NPVs and 1 group II NPVs) and 1 betabaculovirus using the CGView Comparison Tool (CCT) [107]. Moreover, the multiple alignment of conserved genomic sequence with rearrangements was performed by Mauve [108].

## Additional files

Additional file 1: **Table S1.** Characteristics of current sequenced baculovirus genomes. ^1^n/a = no information is available in either the paper or GenBank file. ^2^The GenBank file with accession number KX1513952 is not available at the GenBank website. ^§^Virus name printed in bold were used for further comparison. **Table S2.** ORFs predicted in the TraeNPV genome. **Table S3.** ORF numbers of the 37 baculovirus core genes from 77 baculoviruses. **Table S4.** Sequencing library statistics. **Table S5.** Primer sets for validating the genome assembly and gap filling. *A total of 103 primer sets were used in this study. The average amplicon size is ca. 1150 bp and the re-sequenced coverage is ca. 1. **Table S6.** Primer sets for validating the gene coding regions. (XLSX 99 kb)

Additional file 2: **Figure S1.** Genome density of TraeNPV compared to 78 sequenced baculoviruses. Genome density = number of ORFs/genome size; ratio of genome density = relative genome density to that of TraeNPV. The number behind the column represents the order of the relative genome density among 79 sequenced baculoviruses. (TIFF 2612 kb)

Additional file 3: **Figure S2.** Heat map of the genome. The heat map identity of the genomes from the species AcMNPV, BmNPV, MaviMNPV, LdMNPV and CpGV (from the outside to the inside) compared to the orthologous ORFs in TraeNPV. The darker the red is, the higher the correlated genomic fragment identity. (TIFF 1135 kb)

Additional file 4: **Figure S3.** In silico Restriction Fragment Length Polymorphism (in silico RFLP) pattern based on the whole genomic sequences of TraeNPV and AcMNPV as cut with *Bam*HI restriction enzyme. (TIFF 179 kb)

Additional file 5: **Figure S4.** Flowchart of bioinformatics analysis. (TIFF 711 kb)

## Abbrevations

NGS: Next-generation sequencing
NPV: Nucleopolydedrovirus
ORF: Open reading frame
PE: Paired end
