# Supplementary figures and images for: Genomic sequencing of Troides aeacus nucleopolyhedrovirus (TraeNPV) from golden birdwing larvae (Troides aeacus formosanus) to reveal defective Autographa californica NPV genomic features
Source: BMC Genomics. 2019 May 27;20:419. doi: 10.1186/s12864-019-5713-2 (PMC6537400; doi:10.1186/s12864-019-5713-2)

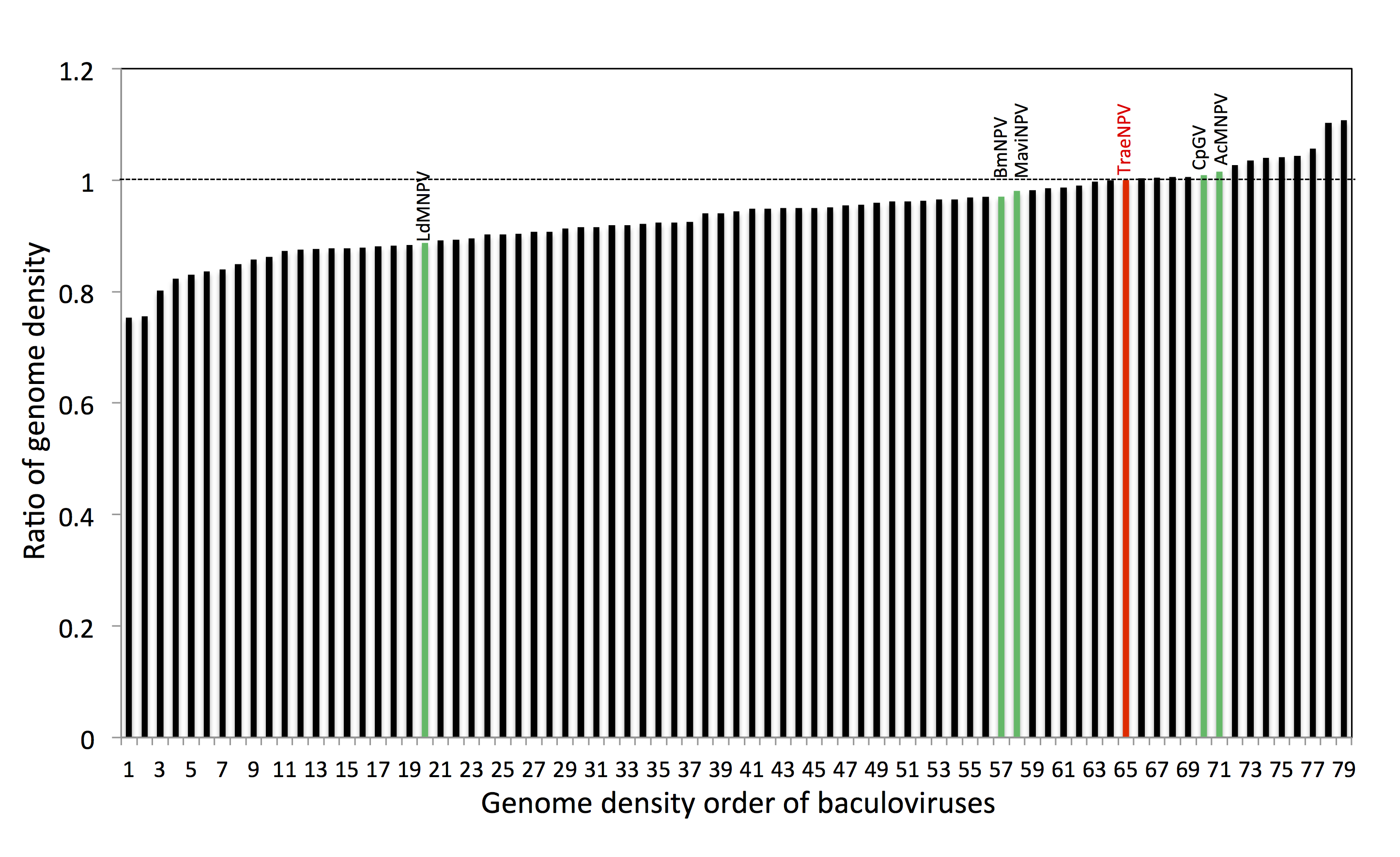

Supplement: Supplementary file 2 — Figure S1. Genome density of TraeNPV compared to 78 sequenced baculoviruses. Genome density = number of ORFs/genome size; ratio of genome density = relative genome density to that of TraeNPV. The number behind the column represents the order of the relative genome density among 79 sequenced baculoviruses. (TIFF 2612 kb) [file 12864_2019_5713_MOESM2_ESM.tiff]

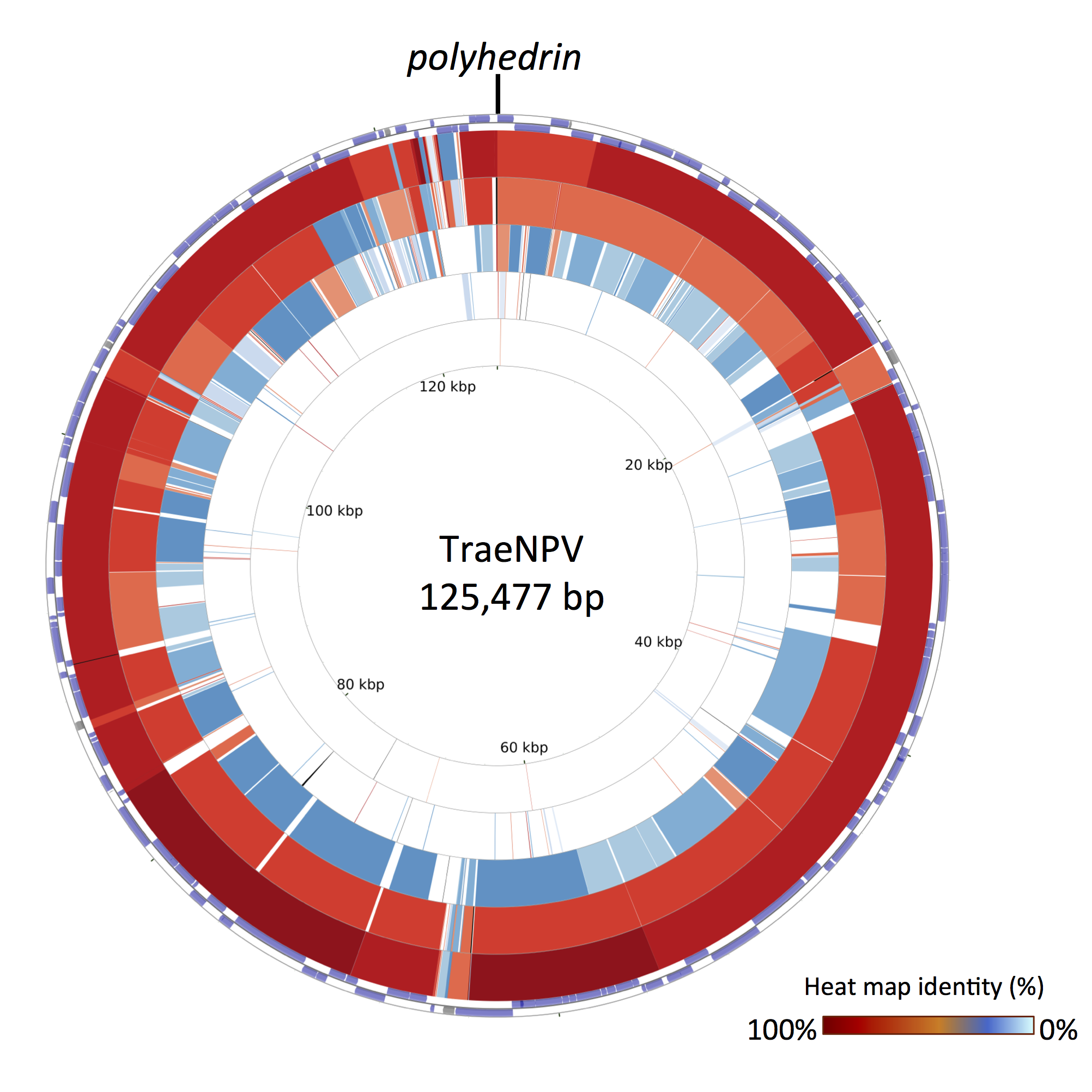

Supplement: Supplementary file 3 — Figure S2. Heat map of the genome. The heat map identity of the genomes from the species AcMNPV, BmNPV, MaviMNPV, LdMNPV and CpGV (from the outside to the inside) compared to the orthologous ORFs in TraeNPV. The darker the red is, the higher the correlated genomic fragment identity. (TIFF 1135 kb) [file 12864_2019_5713_MOESM3_ESM.tiff]

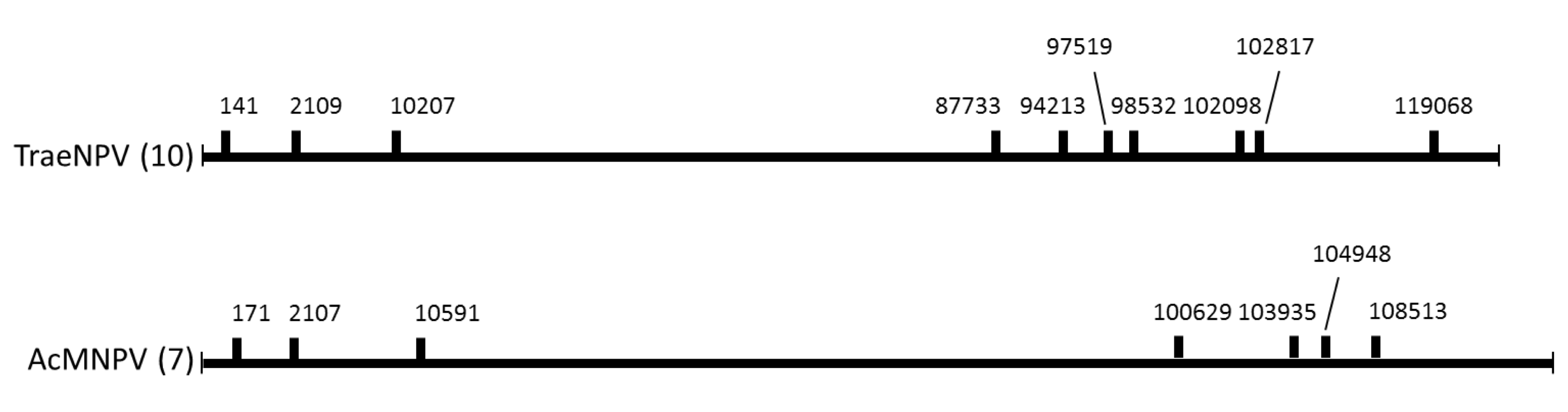

Supplement: Supplementary file 4 — Figure S3. In silico Restriction Fragment Length Polymorphism (in silico RFLP) pattern based on the whole genomic sequences of TraeNPV and AcMNPV as cut with BamHI restriction enzyme. (TIFF 179 kb) [file 12864_2019_5713_MOESM4_ESM.tiff]

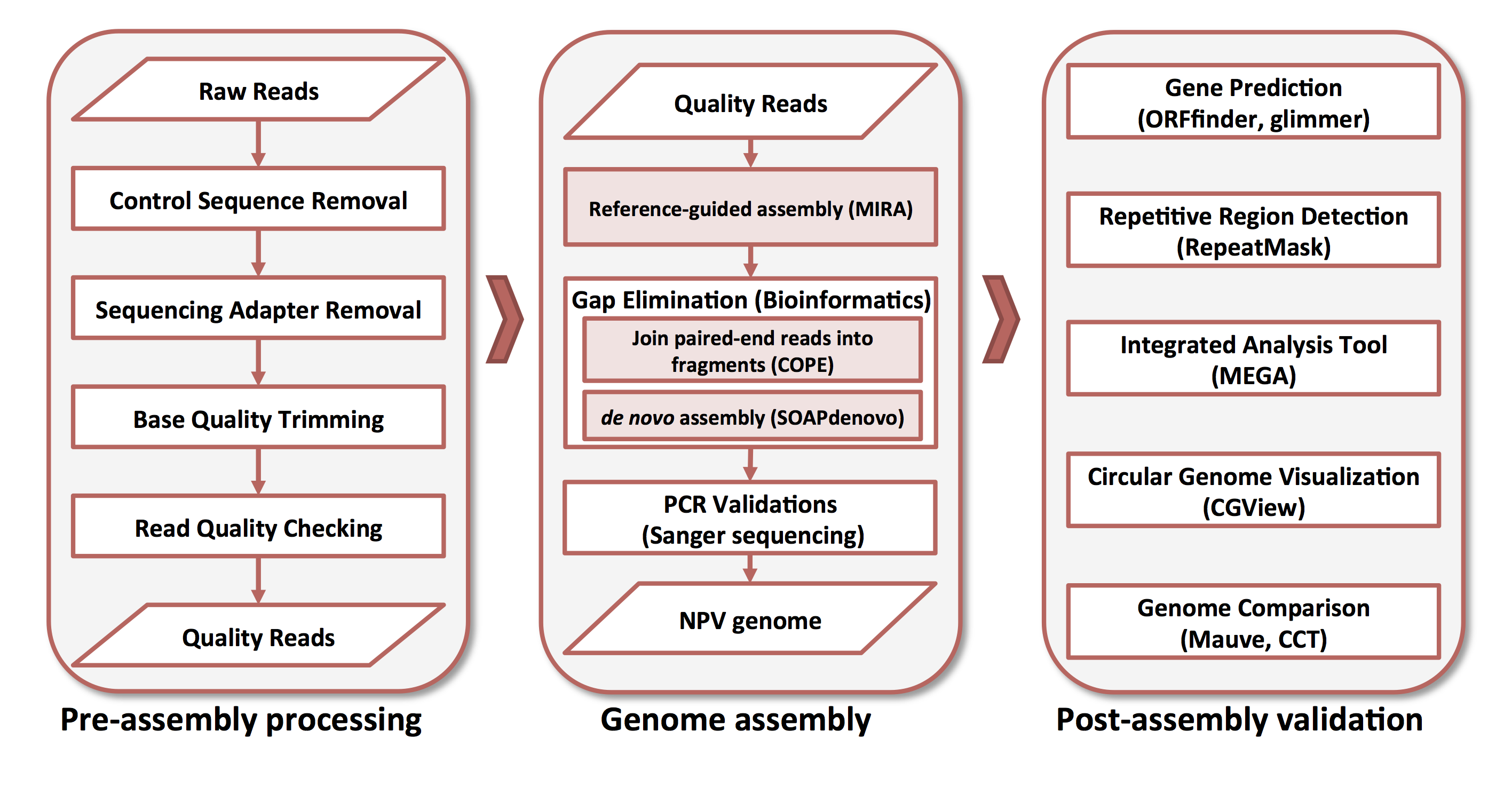

Supplement: Supplementary file 5 — Figure S4. Flowchart of bioinformatics analysis. (TIFF 711 kb) [file 12864_2019_5713_MOESM5_ESM.tiff]
